# Si/SiO_2_/Al_2_O_3_ Supported Growth of CNT Forest for the Production of La/ZnO/CNT Photocatalyst for Hydrogen Production

**DOI:** 10.3390/ma15093226

**Published:** 2022-04-29

**Authors:** Muhammad Irfan, Shazia Shukrullah, Muhammad Yasin Naz, Irshad Ahmad, Bilal Shoukat, Stanislaw Legutko, Jana Petrů, Saifur Rahman, Mabkhoot A. Alsaiari

**Affiliations:** 1 Electrical Engineering Department, College of Engineering, Najran University Saudi Arabia, Najran 11001, Saudi Arabia; miditta@nu.edu.sa (M.I.); srrahman@nu.edu.sa (S.R.); 2 Department of Physics, University of Agriculture Faisalabad, Faisalabad 38040, Pakistan; irshadmahar563@yahoo.com (I.A.); bilalshoukat090@gmail.com (B.S.); 3 Faculty of Mechanical Engineering, Poznan University of Technology, 3 Piotrowo Street, 60-965 Poznan, Poland; stanislaw.legutko@put.poznan.pl; 4 Department of Machining, Assembly and Engineering Metrology, VSB Technical University of Ostrava, 17. Listopadu 2172/15 Street, 70833 Ostrava, Czech Republic; jana.petru@vsb.cz; 5 Empty Qaurter Research Unit, Chemistry Department, College of Science and Art at Sharurah, Najran University Saudi Arabia, Najran 61441, Saudi Arabia; mabkhoot.alsaiari@gmail.com

**Keywords:** zinc oxide, carbon nanotube forest, lanthanum, photocatalytic activity, hydrogen production

## Abstract

The use of ZnO as a photocatalyst with a reduced recombination rate of charge carriers and maximum visible light harvesting remains a challenge for researchers. Herein, we designed and synthesized a unique La/ZnO/CNTs heterojunction system via a sol–gel method to evaluate its photocatalytic performance for hydrogen evolution. A ferrocene powder catalyst was tested for the production of CNT forests over Si/SiO_2_/Al_2_O_3_ substrate. A chemical vapor deposition (CVD) route was followed for the forest growth of CNTs. The La/ZnO/CNTs composite showed improved photocatalytic efficiency towards hydrogen evolution (184.8 mmol/h) in contrast to 10.2 mmol/h of pristine ZnO. The characterization results show that promoted photocatalytic activity over La/ZnO/NTs is attributed to the spatial separation of the charge carriers and extended optical absorption towards the visible light spectrum. The optimum photocatalyst shows a 16 h cycle performance for hydrogen evolution. The H_2_ evolution rate under visible light illumination reached 10.2 mmol/h, 145.9 mmol/h and 184.8 mmol/h over ZnO, La/ZnO and La/ZnO/CNTs, respectively. Among the prepared photocatalysts, ZnO showed the lowest H_2_ evolution rate due to the fast recombination of electron–hole pairs than heterojunction photocatalysts. This research paves the way for the development of ZnO and CNT-based photocatalysts with a wide optical response and reduced charge carrier recombinations.

## 1. Introduction

ZnO is a semiconductor photocatalyst with high oxidative potential, excellent photosensitivity, moderate stability, and variable band structure, which has sparked widespread interest in photocatalytic fuel production and environmental remediation [1]. Pristine ZnO, however, does not respond well to the visible part of the light spectrum when used as a photocatalyst. The photocatalytic activity of pristine ZnO is also hampered by the rapid recombination of excitons and photocorrosion [2]. These issues can be addressed by incorporating rare-earth oxides and carbonaceous materials into ZnO. Rare-earth metals are recognized for their large surface area and high charge carrier trapping ability, which can greatly reduce the recombination of excitons. The greater ionic radius of rare-earth ions than Zn^2+^ ions is expected to cause lattice distortions in ZnO, increasing the lattice constant and reducing the ZnO band gap. According to some studies, the rare-earth element-doped ZnO lattice expands due to the formation of structural defects in the form of oxygen vacancies. These vacancies work as charge trap centers and prolong the recombination time for charge carriers [3,4]. The rare-earth elements in the structure boost the electron mobility by producing extra charge carriers when Zn^2+^ is replaced with rare-earth ions. The carbonaceous material, on the other hand, produces defective Zn-O-C bonds in the lattice to bring a change in the activation range of ZnO from the ultraviolet to visible light spectrum. CNTs, in this regard, are captivating the attention of the research community because of their remarkable physical, thermal, electrochemical, and mechanical characteristics [5].

CNTs can be employed as a support material for producing efficient photocatalysts’ materials for photocatalytic hydrogen evolution and the treatment of the environment. CNTs show a larger surface area, high electrical and thermal stability and excellent charge mobility. CNTs can be utilized as a photosensitizer in a photocatalyst design to generate a large number of electrons during photocatalytic reactions. These electrons jump from the valance band to the conduction band to expedite the reaction rate by reducing the energy band gap of the photocatalyst [6]. At the same time, CNTs act as an electron sink. The electrons become trapped in CNT structure and therefore do not spontaneously return to the valance band. Oliveira et al. [7] revealed the better photocatalytic performance of ZnO/CNTs photocatalyst than the pristine ZnO probably due to a shift in the absorption light range. The photocatalytic activity can be further improved by combining rare-earth elements with ZnO/CNTs system. For example, high photocatalytic activity is possible by doping ZnO with Nd and compositing with CNTs. The Ag–ZnO@Fe_3_O_4_/CNTs and ZnO/Zn–C nanofiber type composites are also reported for high photocatalytic performance under visible light irradiation [8]. The silver and Zn–C nanofibers in the composite cause a red shift in absorption by forming intermediate energy levels, which trap the charge carriers to enhance the photocatalytic performance. Similarly, a synergism effect of cationic doped ZnO and CNTs also improves the light absorption and delays the recombination of charges, as reported by Toloman et al. [9]. Similarly, Elias et al. [10] doped ZnO with V and La to produce a V-La/ZnO/CNTs photocatalyst for hydrogen production. They reported higher photocatalytic activity for the composite than the pristine ZnO. 

In photocatalytic H_2_ evolution over a La/ZnO/CNT photocatalyst under light irradiation, photoinduced electrons in ZnO migrate from the valance band to the conduction band, leaving holes in the valance band. These electrons in the conduction band are captured by La ions and thus transported to the surface of ZnO. Meanwhile, due to their role as photosensitizers and electron sinks, conduction band electrons flow towards CNTs, accelerating the transport of electrons to the surface of ZnO. This action of CNTs enhances the separation between the chare carriers. The electrons in the conduction band interact with H^+^ to form H_2,_ whereas holes in the valance band are scavenged by the sacrificial reagent. As a result of the synergy between CNTs, La, and ZnO, the creation of La/ZnO/CNTs composite causes improved the separation of photoinduced charges, which enhances H_2_ evolution under light irradiation.

The performance of CNT support in a composite photocatalyst largely relies on its structural formation and purity. Most of the previously reported work on composite photocatalysts was carried out by taking commercially available CNTs without studying their purity and structural defects. The lack of information on the structural parameters minimizes the role of CNTs in photocatalytic activity. CNTs’ structures can be produced through different routes such as arc discharge, the laser breakdown of metals, hydrothermal method, plasma discharge method, chemical vapor deposition [11,12,13]. CVD is the best and most popular technique for producing high-quality CNTs. Being the simplest and most economical method of production of CNTs, CVD is mostly used for the large-scale production of CNTs [14]. This process is considered the most efficient synthesis method of CNTs, both qualitatively and quantitatively. Baddour and Briens [15] reported that catalytic techniques such as CVD are relatively easier to execute since these have a low production cost and are less energy-intensive. José-Yacamán et al. [16] first developed a catalytic CVD method to produce CNTs. In this method, CNTs are produced by using catalytic materials to decompose carbon-containing molecules. Ethylene, ethanol, methane, hexane, anthracite, carbon monoxide, naphthalene, carbon dioxide, benzene, and acetylene are widely used carbon precursors. The method of electrical or thermal energy splits the molecule into atomic carbon, which shows high reactivity. Several specific process variables, such as metal catalysts, catalyst support, source and concentration of hydrocarbons, flowrates, temperature and time, among others, significantly affect the structural properties and yield of the final product. Therefore, it is not an easy task to find suitable conditions in which the CNT material of specific characteristics can be produced [17].

In the reported work, a forest of CNTs was grown over Si/SiO_2_/Al_2_O_3_-supported ferrocene catalyst by considering a catalytic CVD method. The produced CNTs were ultrasonically processed, characterized for the structural formation and finally used to produce the La/ZnO/CNT composite for hydrogen production. There is no publication on La/ZnO/CNT employing Si/SiO_2_/Al_2_O_3_ as a support material for CNT production. Moreover, the prepared photocatalyst showed remarkable stability for H_2_ evolution.

## 2. Materials and Methods

### 2.1. Production of CNT Forest

**Figure 1:**
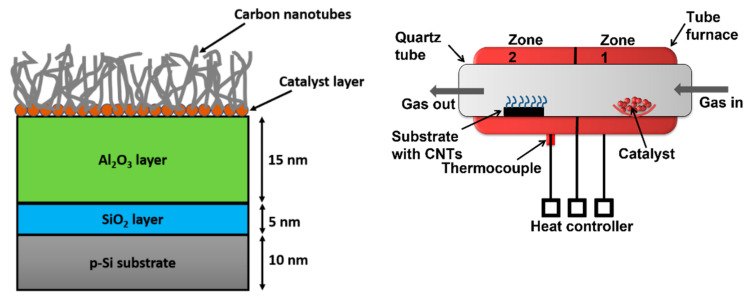
Illustration of the steps involved in the fabrication of Si/SiO_2_/Al_2_O_3_ substrate and CVD grow of CNT forest.

In the first step, silicon/silica/alumina (Si/SiO_2_/Al_2_O_3_) substrates were prepared by taking 2 cm × 2 cm pieces of the silicon wafer. The thickness of the wafer was 10 nm. These p-type silicon (100) wafers were cleaned using a wet cleaning technique. It is a standard cleaning technique used to clean silicon wafers before performing oxidation, diffusion and CVD processes. As illustrated in Figure 1, the dry thermal oxidation of the silicon (Si) was performed to grow a 5 nm-thick silica (SiO_2_) layer on Si wafer. For thermal oxidation, Si wafers were placed in a tube furnace. Since vertical tube furnaces are higher than horizontal tube furnaces, some micro-fabrication setups have difficulty using vertical furnaces. Therefore, a horizontal furnace was used to prepare the substrates. The furnace temperature was increased to 1100 °C in an argon-rich environment. Dry thermal oxidation is usually performed in the range of 800–1200 °C. After gaining the desired temperature, the oxygen gas was supplied in the tube for 4 h. Since silicon is reactive to oxygen, an amorphous SiO_2_ layer is formed immediately after the reaction between silicon and oxygen. The molecular oxygen works as an oxidant in the following oxidation reaction: Si + O_2_ → SiO_2_. The layered substrate was cooled down under argon flow. A SiO_2_ layer of Si substrate eliminates Si effectiveness and outlines the growth of CNTs.

A 15 nm film of Al_2_O_3_ was grown over the SiO_2_ layer. The Al_2_O_3_ buffer layer was grown over the substrate using an evaporation process. The beam evaporation is a low-cost, simple layer deposition technology for growing well-crystallized oxide layers. The post-deposition annealing of the substrate was performed at 600 °C. This multi-layered substrate structure with an SiO_2_ layer sandwiched between the upper Al_2_O_3_ layer and lower Si was used to grow MWCNTs. Ferrocene was used as a catalyst for CNT forest growth over the Si/SiO_2_/Al_2_O_3_ substrate. The optimized CVD conditions, as reported in our previous study, were used to produce a forest of MWCNTs. 

A split zone tube furnace was used in the CVD method as illustrated in Figure 1. The substrate was placed in zone-2 while the Fe(C_2_H_5_)_2_ catalyst was placed in zone-1. The precursor and carrier gases were passed from zone-1 into zone-2 at 800 °C temperature. Under these heating conditions, Fe(C_2_H_5_)_2_ particles turned into Fe particles, which work as a catalyst seed for cracking hydrocarbon (ethylene) into graphitic carbon. The process temperature was raised to the desired level under an argon gas flowrate of 40 sccm. Ethylene and hydrogen were also supplied into the tube at set flowrates of 60 sccm and 100 sccm. The ethylene molecules cracked into hydrogen and carbon over Fe particles. The carbon atoms deposit over the substrate and grow into forest form. The forest growth was sustained for one hour. The nanotubes were removed from the substrate by performing sonication for 30 min. The surface morphology, purity, internal structure and surface defects of CNTs were studied before being used in the synthesis of a composite photocatalyst.

### 2.2. Characterization of Samples

X-ray diffraction analysis of the prepared samples was carried out to analyze their structural phase development, lattice parameters, structural defects and crystallite size. An X-ray diffractometer (Bruker D8 XRD) was used to produce diffraction patterns, operated with a 40 kV source. A Cu–Kα radiation source with a wavelength of 1.54 Å was used as an X-rays beam. The photoluminescence analysis was conducted with an instrument model iDus-420 to determine the band gap energy and luminescence of the pure and composite photocatalysts. A scanning electron microscope (Nova-Nano) was used to produce images of surface morphology of the produced photocatalysts. A double beam UV–Vis spectrophotometer (CE-7200) was employed to produce the UV spectra of the adsorbents. A TGA/DSC instrument was used to obtain the thermogravimetry profiles of the samples in the temperature range of 100–900 °C. An RMP-510 Raman Spectrometer was used to produce the Raman spectra of the samples.

### 2.3. Production of La/ZnO/CNTs Photocatalyst

A simple and inexpensive sol–gel route was followed to prepare La/ZnO/CNTs composite as follows: 30 g of lanthanum acetate and zinc acetate were dispersed in 130 mL of DI water and 0.3 g of as-prepared carbon nanotubes were added to the mixture solution for the fabrication of La/ZnO/CNTs composite. The carbon nanotubes were first functionalized in an aqueous solution of sulfuric acid and nitric acid in a ratio of 4:1 owing to the insolubility of carbon nanotubes in organic solvents because of their stable structure and carbon element. The solution was magnetically stirred at 90 °C for 1 h to attain the obvious sol with a dropwise addition of ammonia to reach the solution pH of 9. Afterward, the aged particles were washed with ethanol and DI water and centrifuged to wipe out any traces of impurity. To obtain the final product for the characterization and photocatalytic tests, the as-obtained product was heat-treated in an industrial oven at 120 °C for 12 h followed by calcination at 700 °C for 3 h.

### 2.4. Hydrogen Evolution Experiments

**Figure 2:**
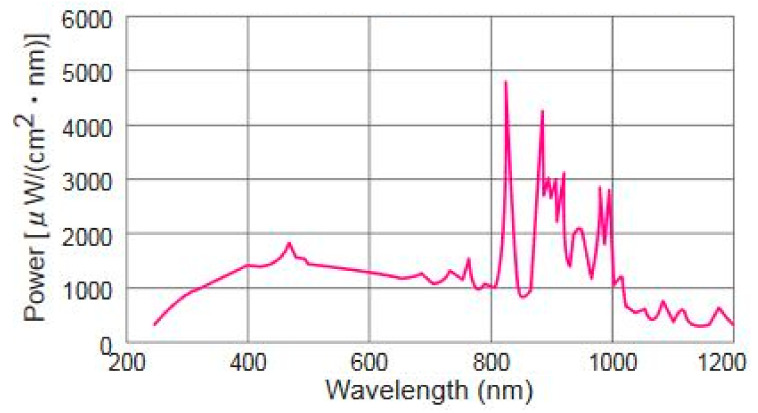
The spectral intensity of Xe light source.

Using a water–glycerol combination, the hydrogen production efficiency of the as-prepared ZnO-based photocatalysts was tested under visible light exposure. The H_2_ evolution tests were conducted using a heat-free 300 W Xe light source fitted with a UV filter and positioned at 0.012 m away from the quartz reactor. The typical spectral intensity plot of the Xe light is given in Figure 2. This information was extracted from the link: https://www.gmp.ch/htmlarea/pdf/asahi_pdf/max302.pdf (accessed on 24 April 2022). To conduct a photocatalytic reaction, 25 mg of the as-prepared sample was magnetically mixed in 80 mL aqueous solution containing 10% of glycerol and stirred to obtain the uniformity of the solution. The hydrogen production in the photocatalytic reaction was assessed after each hour of the light exposure.

## 3. Results and Discussion

### 3.1. Structural Formation and Surface Morphology

**Figure 3:**
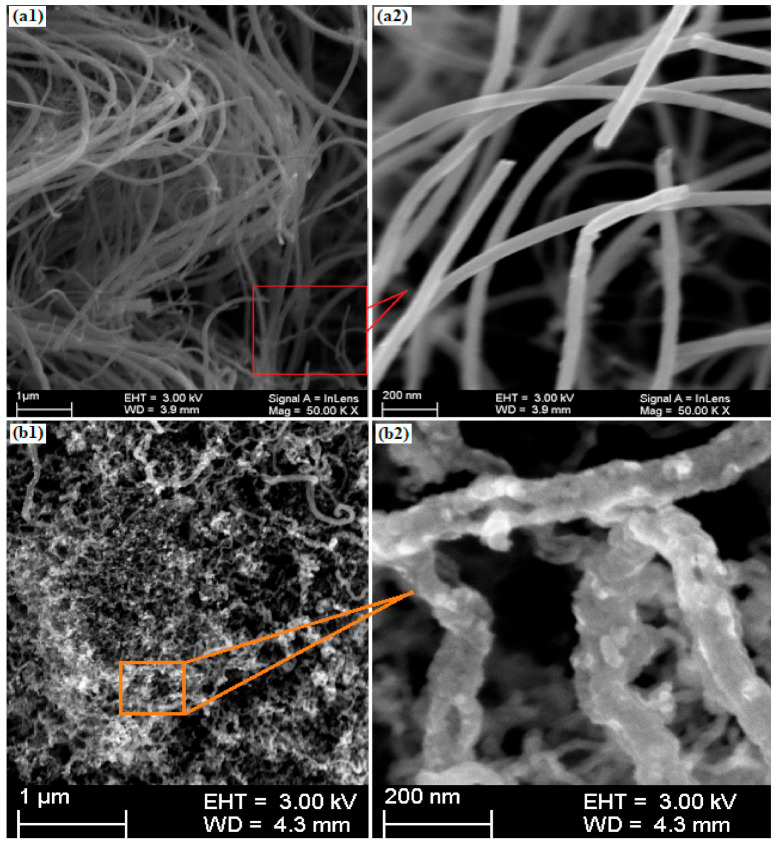
(**a1**) SEM image of the CNT forest; (**a2**) magnified SEM image of CNTs removed from the substrate through sonication; (**b1**) SEM image of La/ZnO/CNTs composite photocatalyst; and (**b2**) magnified SEM image of La/ZnO/CNTs composite photocatalyst showing the deposition of ZnO and La particles on the tube structure.

The SEM micrograph of the CNT forest is shown in Figure 3(a1), which reveals the dense and uniform growth of CNT structures. The highly magnified SEM micrograph of the forest is shown in Figure 3(a2). The fine structures with uniform diameter distribution are evident in the magnified micrograph. Some microscopic particles were also found in the CNT forest, representing catalyst particles dissolved in the CNTs during sonication. CNTs were the least entangled and most discernible in the enlarged SEM images. At high magnification, no surface flaws, sharp edges, branching formations, or end-capping were seen. The tube length varied from a few micrometers to several micrometers. Danafar et al. [18] employed various ferrocene particle sizes and weights to create CNTs. They discovered that the catalyst weight influences both the amount and the structural development of nanotubes. However, their research found an inverse link between the particle size and formation rate. A high surface area-to-volume ratio is responsible for increased formation rate as particle size decreases. For the same catalyst weight, the reactive surface area increases as the particle size decreases. The catalyst’s catalytic activity improves as the reactive surface area increases, as does the yield and structure formation.

The SEM images of the as-prepared Al/ZnO/CNTs photocatalyst clearly show that La and ZnO particles were grown on the surface of CNTs. The presence of CNTs promoted the abundance of catalytic sites and thereby provided the intimate contact between La and ZnO to accelerate the photocatalytic activity. Moreover, based on previous studies, the intimate contact between ZnO and CNTs can increase the transmission and separation of charge carriers to support the photocatalytic H_2_ evolution activity.

**Figure 4:**
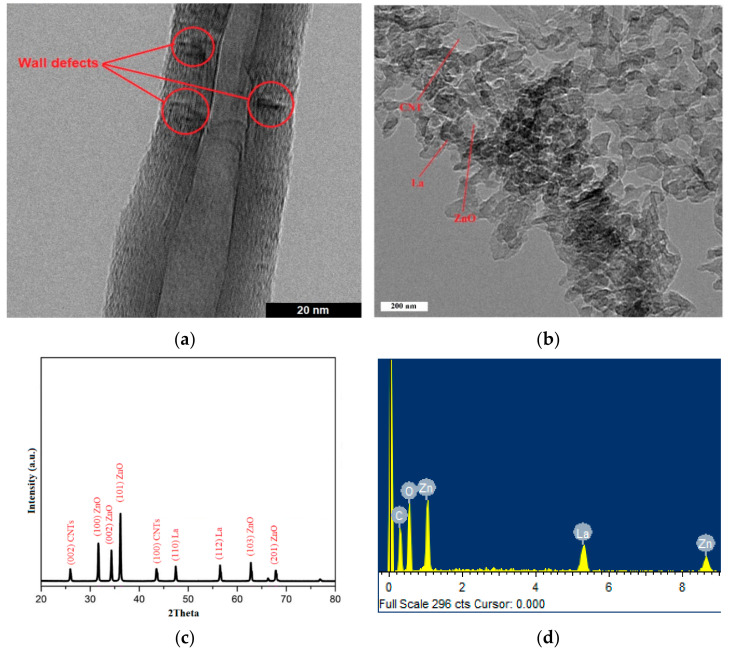
(**a**) TEM morphology of pristine CNTs; (**b**) TEM morphology of La/ZnO/CNTs composite; (**c**) XRD pattern of La/ZnO/CNTs composite; and (**d**) EDX pattern of La/ZnO/CNTs composite photocatalyst.

Figure 4a,b show TEM images of a nanotube with wall defects and La/ZnO/CNTs composite, respectively. The structural planes and chemical composition of the La/ZnO/CNTs composite are shown in Figure 4c,d, respectively. These analyses clearly show the deposition of ZnO and La particles on the CNT structure. The underlying morphology and multi-layered creation of CNT structures was explored by high-resolution TEM scans. The inner bore diameter, exterior bore diameter, number of concentric layers, and distance between subsequent layers may all be measured. The internal bore and outside tube diameters were almost identical throughout the tube’s length. Impurity particles, most likely catalyst particles or defects in the multiwalled growth, were also found in the tube structures. During the diffusion and precipitation stages, some particles may have been trapped by the carbon. The over-precipitation of carbon at various time periods during the formation of structures is responsible for the dark patches seen inside the concentric layers. This demonstrates that structural defects may be reduced by adjusting the catalyst weight.

The spacing between consecutive layers remained between 0.34 nm and 0.35 nm [19]. The smallest spacing of 0.34 nm was measured in CNT structures. Smaller spacing reflects the close packing of multilayers within individual structures and reduced structural defects. The electrical and mechanical character of CNTs also increases with close packing. The average diameter of CNT structures was measured to be approximately 32–38 nm. Lanzani et al. [20] reported that the encapsulation of seed particles by the tube results in an increase in tube diameter and structural defects. Hayashi et al. [21] used a similar substrate to grow CNT forests using thermal CVD at 700 °C. They used Al_2_O_3_-supported Fe catalyst and acetylene/hydrogen/nitrogen gas mixture to produce CNT structures. They were able to grow a CNT forest of 0.6 mm height. They also reported that the initial disorder and seed trapping in structures discontinue the formation of continuous CNT structures before reaching the maximum height. The tube thickness increased with the size of catalyst seed. Augiar et al. [22] also used a similar substrate to grow CNT structures. They stated that the nature of the interaction between the substrate and catalyst seeds is also of critical importance in the growth mechanism. However, this mechanism is not fully understood yet.

The XRD pattern of La/ZnO/CNTs is shown in Figure 4c, which confirms the hexagonal wurtzite phase of ZnO as matched with JCPDs 89-0510. The XRD peaks at 2θ values of 31.73, 34.36 and 36.18 belong to principal planes (100), (002) and (101) of ZnO. Furthermore, the XRD pattern of the La/ZnO/CNTs composite photocatalyst shows two more diffraction peaks of CNTs at 2θ of 26.14° and 43.31°, which correspond to (002) and (100) crystal planes, respectively. The diffraction peak at 2θ of 26.14° indicates C (002) reflection of the graphite structure of CNTs. The crystallite size, determined by the Scherrer formula, was roughly 34 nm and 26 nm for ZnO and La/ZnO/CNTs, identifying the effective role of La and CNTs in suppressing the crystal growth of ZnO. The smaller crystallite size can increase the surface area to provide more active sites to accelerate the photocatalytic activity. The EDX spectrum of the La/ZnO/CNTs composite is depicted in Figure 4d, where Zn, O, La and C elements were identified as the main contributing elements. No trace elements or impurities were found, revealing the successful synthesis of La/ZnO/CNTs. 

### 3.2. Structural Purity and Weight Loss Analysis

**Figure 5:**
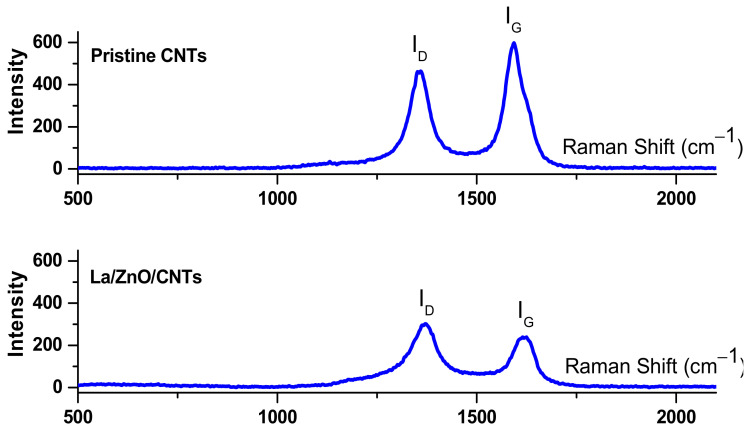
Raman spectra of pristine CNTs and La/ZnO/CNTs composite photocatalyst.

Raman spectroscopy and TGA analyses were used to evaluate the crystallinity and weight loss of pristine CNTs and La/ZnO/CNTs composite photocatalyst. Raman spectra provide two important bands, namely the D-band and G-band. An intense D-band indicates high structural defects and low crystallinity of CNT structures. Conversely, an intense D-band is an indication of the growth of highly crystalline structures [23]. Figure 5 shows the Raman spectra of pristine CNTs and La/ZnO/CNTs composite photocatalyst. These spectra show that the D-band appears in the Raman shift range of 1344–1364 cm^−1^ while the G-band appears in the Raman shift range of 1571–1608 cm^−1^. The G-bands near 1583 cm^−1^ for pristine CNTs are sourced by the C–C bond starching of graphite. This bond starching is common in sp^2^ hybridized carbon systems. If the ratio of intensities of the defective and graphitic band (I_D_/I_G_) is low, the structure would be more graphitic. The I_D_/I_G_ ratio of pristine CNTs and La/ZnO/CNTs composite photocatalyst was measured to be approximately 0.71 and 0.85, respectively. This ratio indicates better the structural growth of pristine CNTs and high surface roughness in the La/ZnO/CNTs composite due to the deposition of La and ZnO particles on the tube structure. The surface roughness and structural defects are generally supportive of high photocatalytic activity due to the production and trapping of the electrons.

**Figure 6:**
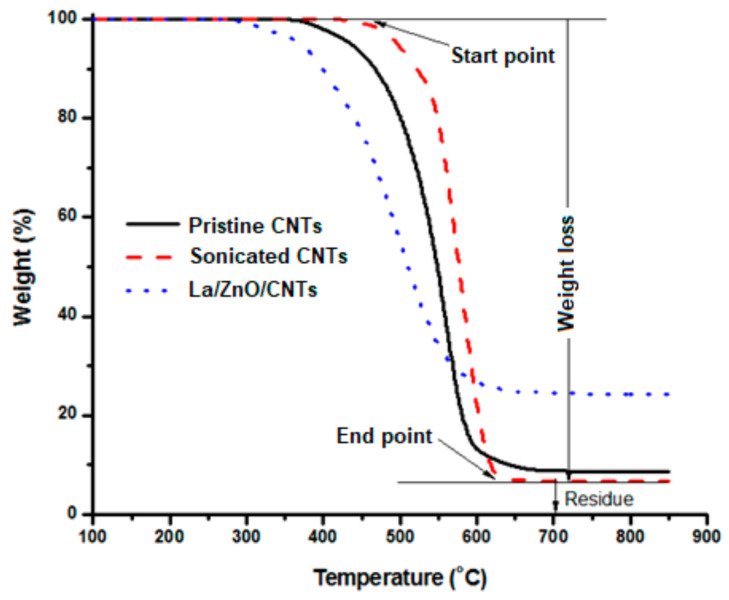
TGA profiles of pristine CNTs, sonicated CNTs and La/ZnO/CNTs composite photocatalyst.

Figure 6 shows the TGA profiles of pristine CNTs, sonicated CNTs, and La/ZnO/CNTs composite photocatalyst. TGA profiles are used to assess the purity of nanostructures. The changes in TGA profiles are marked by onset point, weight loss, and endpoint. The onset point is the temperature at which the sample starts losing weight. The endpoint is the temperature from which the sample did not lose any further weight. The percentage weight below this curve shows the presence of impurities in the sample in the form of residue. The percentage weight lost between the onset and endpoints shows the percentage purity of CNT structures. Sometimes, there are some variations in the TGA profile just before reaching the onset point temperature, which shows the presence of moisture in the sample. In this study, no moisture content was observed in the CNT structures. The maximum weight loss was measured for sonicated CNTs, followed by pristine CNTs and La/ZnO/CNTs composite photocatalyst. The large weight loss is referred to the high purity of the structures.

The residual of sonicated CNT structures was approximately 6%, indicating the 94% purity of the nanostructures. The catalyst particles, amorphous carbon, and/or alumina particles, which appeared during the removal of the nanotubes from the surface of the substrate, may be present in the residue. The onset point is the start of the decomposition of the sample and the point where the sample decomposes completely is called the endpoint. Below this point, only impurities were left in the sample while CNT structures were completely decomposed. The published literature shows that the endpoint of multiwalled CNT structures falls within 400 °C and 650 °C while the endpoint of single-walled CNT structures and amorphous carbon falls within 350 °C and 500 °C and 200 °C and 300 °C, respectively [24]. The endpoint temperature of the reported structures remained between 540 °C and 590 °C, which confirms the formation of a multiwalled CNTs-based composite photocatalyst.

### 3.3. Cyclic Voltammetry of Photocatalyst

**Figure 7:**
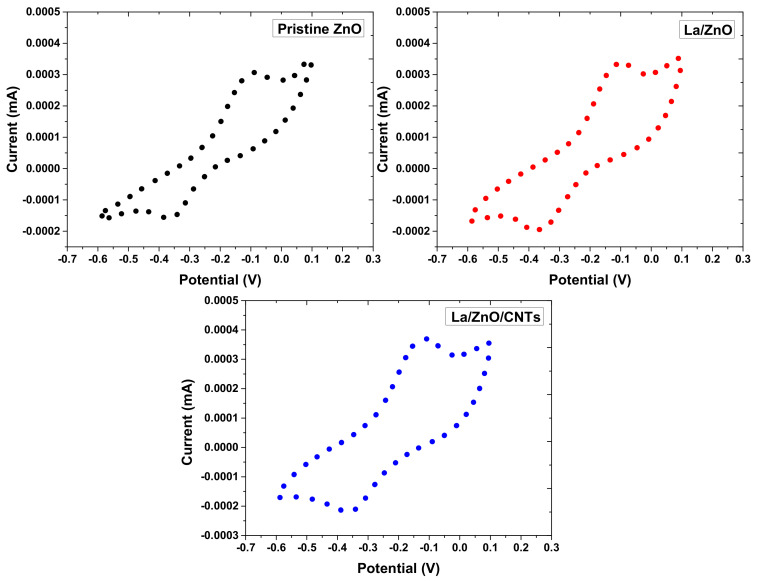
Cyclic voltammetry profiles of pristine ZnO, La/ZnO, and La/ZnO/CNTs composite photocatalyst.

The PL analysis revealed a typical ZnO emission band in the UV region due to electron–hole recombinations. The PL emissions from La/ZnO and La/ZnO/CNTs showed a shift towards a longer wavelength than pure ZnO, identifying a decrease in the optical band gap to assist the photocatalytic process. The PL emission intensity of the UV band decreased in the order of ZnO > La/ZnO > La/ZnO/CNT. Usually, the smaller PL emission intensity of the sample reveals the greater suppression of charge carriers. Obviously, the combined effect of La and CNTs effectively reduces the recombination of charge carriers, which can be helpful in boosting photocatalytic hydrogen evolution activity. CV analysis was carried out to confirm the efficient transmission of charge carriers over as-prepared photocatalysts, and the results are shown in Figure 7. It is evident that pure ZnO showed the smallest anodic and cathodic peak current, which identifies its poor charge transportability. The La/ZnO/CNTs composite showed the highest anodic and cathodic peaks current to confirm the swift charge transfer due to the effective role of La and CNTs. Therefore, the CV results clearly show that the enhanced transfer of charge carriers on the surface of La/ZnO/CNTs, in contrast to bare ZnO, can be assigned to the promoted charge transmission capability of CNTs. Moreover, enhanced current with lower peak-to-peak potential strongly confirms the decreased interfacial resistance for charge migration in redox reactions.

**Figure 8:**
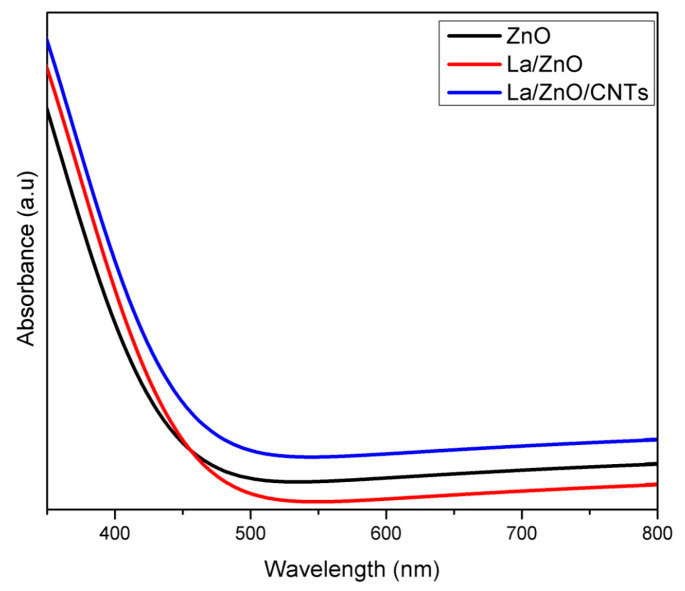
Optical absorption spectra of pristine ZnO, La/ZnO, and La/ZnO/CNTs composite photocatalyst.

To study the optical absorption properties, the optical absorption spectra of as-prepared photocatalysts are shown in Figure 8. Compared to pure ZnO, the optical absorption of La/ZnO shifted towards a longer wavelength with an absorption edge at 390 nm. The improved absorption could be attributed to the presence of La ions, which lowered the optical band gap and thereby increased the optical absorption. Similarly, La/ZnO/CNTs showed a further redshift in the optical absorption due to the influential role of CNTs. Therefore, it is obvious that a synergistic effect between La and CNTs significantly increases the optical absorption behavior of ZnO to enhance the photocatalytic activity. 

### 3.4. Hydrogen Evolution

**Figure 9:**
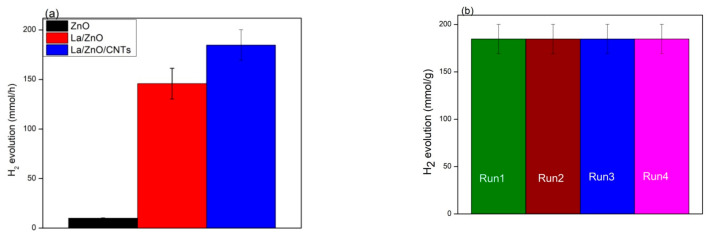
(**a**) H_2_ evolution rates over pristine ZnO, La/ZnO, and La/ZnO/CNTs after 4 h of visible light illumination. Error bars indicate the standard deviation measured by three independent experiments; (**b**) Stability of La/ZnO/CNTs after successive five cycles. The error bar indicates the standard deviation measured by three independent experiments.

Figure 9a reports the photocatalytic H_2_ evolution rate over pristine ZnO, La/ZnO and La/ZnO/CNTs photocatalysts under visible light illumination. It is observed that the H_2_ evolution rate after 4 h of visible light illumination reached 10.2 mmol/h, 145.9 mmol/h and 184.8 mmol/h over ZnO, La/ZnO and La/ZnO/CNTs, respectively. Among the prepared photocatalysts, ZnO showed the lowest H_2_ evolution rate of 10.2 mmol/h, which may be referred to as the rapid recombination of charge carriers. In contrast to pure ZnO, La/ZnO showed a much higher H_2_ evolution rate under visible light illumination. An increase in the H_2_ evolution rate over La/ZnO was attributed to the effective role of La in the ZnO lattice to enhance the separation between electrons and holes, as confirmed through Pl analysis [25,26]. The optimum H_2_ evolution rate was obtained over the La/ZnO/CNTs composite, which identifies the effective role of CNTs in suppressing the recombination of charge carriers and providing abundant active sites. To test the recyclability and stability of the as-prepared La/ZnO/CNTs photocatalyst, the photocatalytic H_2_ evolution rate was measured for five consecutive cycles. Figure 9b shows the H_2_ evolution rate over repeated cycles, which did not decline during reuse. This trend reflects the better stability of the composite photocatalyst. The photocatalyst in the current study performed much better than most of the previously reported heterojunction photocatalysts [2,27]. The improved photocatalytic response of La/ZnO/CNTs is attributed to CNTs. The nanotubes in the composite acted as a photosensitizer and electron sink, accelerating electron transport to the ZnO surface. Nanotubes also suppress the charge recombination by increasing the separation between electrons and holes in pairs [2,28].

### 3.5. Photocatalytic Mechanism

**Figure 10:**
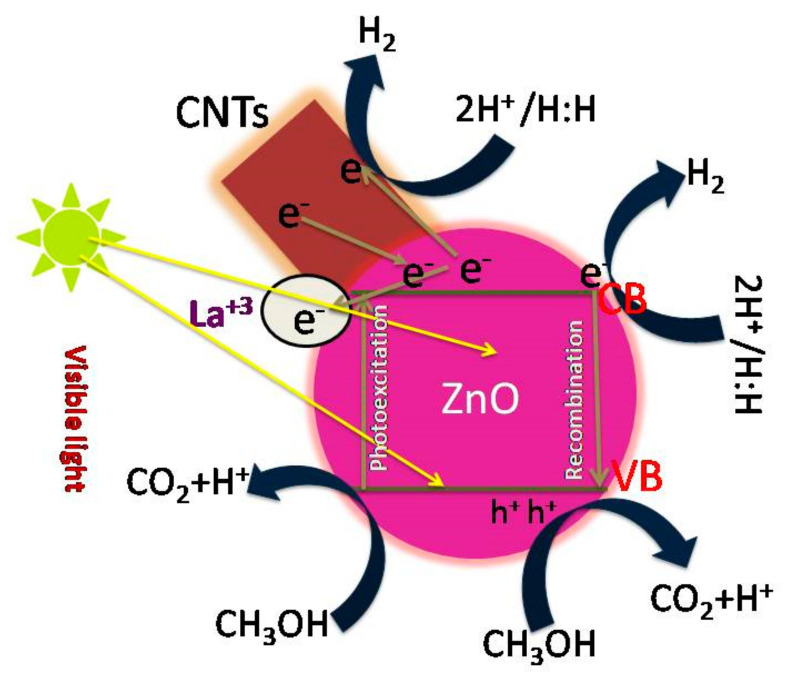
Illustration of the mechanism of H_2_ evolution over the La/ZnO/CNTs photocatalyst.

There might be different reasons relating to the interaction between ZnO and CNTs, such as van der Waals force, π–π bond, and σ^*^ bonds. However, a clear understanding of the physical or chemical interaction between ZnO and CNTs has not arrived yet. This study aimed to explore the role of CNTs in boosting the photocatalytic activity of the La-doped ZnO heterojunction photocatalyst. Figure 10 illustrates the mechanism of photocatalytic H_2_ evolution over the La/ZnO/CNT photocatalyst. The mechanism proposed herein drives the photocatalytic activity of the La/ZnO/CNT composite under sunlight irradiation. Under light exposure, the photoinduced electrons in the valance band of ZnO move to the conduction band, leaving behind holes in the valance band. The electrons in the conduction band are captured by La ions where they move to the surface of ZnO. Since CNTs were added to the composite as a photosensitizer, some of the electrons’ flow conduction of ZnO to CNTs. The photosensitizing and electron sink characteristics of CNTs accelerate the electron transportation to the surface of ZnO by enhancing the separation of electron and hole pairs. Therefore, electrons in the conduction band react with H^+^ ions to produce hydrogen. Similarly, the holes in the valance band were scavenged by the sacrificial reagent. This reveals the enhanced separation of photoinduced charges in La/ZnO/CNTs photocatalyst due to the synergism among CNTs, La and ZnO, which consequently promotes H_2_ evolution under visible light illumination [29].

## 4. Conclusions

A forest-like growth of CNT structures was performed over Si/SiO_2_/Al_2_O_3_ support using a conventional CVD method. Ferrocene was considered a catalyst for the catalytic cracking of ethylene into CNT forest. The carbon product was ultrasonically processed and then used to produce La/ZnO/CNT photocatalyst for hydrogen evolution from a water–glycerol mixture. A set of pristine ZnO, La/ZnO and La/ZnO/CNTs photocatalysts was produced using the sol–gel route and evaluated for photocatalytic activity under visible light illumination. The various characterization tools were used to characterize the structure, morphological, and optical features of the as-prepared samples. Based on PL results, the La/ZnO/CNTs composite showed a reduced recombination and increased transportation of charges due to the combined effect of La and CNTs. Therefore, the prepared La/ZnO/CNTs photocatalyst exhibited the highest H_2_ evolution rate of 184.8 mmol/h under visible light illumination in contrast to 10.2 mmol/h of ZnO. Moreover, the optimum photocatalyst showed strong stability for H_2_ evolution for 15 h. The recyclability and stability of the La/ZnO/CNTs photocatalyst were tested over five consecutive cycles to check the economic value of the process. The hydrogen evolution rate did not decline during the multiple cycling process. These findings suggested the high stability and low process cost of the tested composite photocatalyst. The present study can promote the photocatalytic interest in designing novel photocatalysts based on ZnO and CNTs.

## Data Availability

The reported data is available from the corresponding authors on a reasonable request.
